# Prediction of Moderate to Severe Obstructive Sleep Apnea Using Neck Computed Tomography With Computational Fluid Dynamics Study

**DOI:** 10.3389/fmed.2022.838367

**Published:** 2022-02-03

**Authors:** Wei-Sheng Chung, Sunny Chung

**Affiliations:** ^1^Department of Internal Medicine, Taichung Hospital, Ministry of Health and Welfare, Taichung, Taiwan; ^2^Department of Health Services Administration, China Medical University, Taichung, Taiwan; ^3^Department of Healthcare Administration, Central Taiwan University of Science and Technology, Taichung, Taiwan; ^4^Department of Chemistry, Point Loma Nazarene University, San Diego, CA, United States

**Keywords:** obstructive sleep apnea, overnight oxygen desaturation, computed tomography, computational fluid dynamics, airway pressure, airflow velocity

## Abstract

**Background:**

Moderate to severe obstructive sleep apnea (OSA) is associated with cardiovascular disease. Polysomnography is time intensive and difficult to access for diagnosis of OSA. Neck computed tomography (CT) provides upper airway delineation but not diagnostic criteria for moderate to severe OSA. We explored neck CT with computational fluid dynamics (CFD) study for airway pressure and airflow velocity to predict moderate to severe OSA.

**Methods:**

Enrolled from February 1, 2020, to June 30, 2021, patients with OSA with overnight oxygen desaturation (sPO2 <90%) received awake neck CT with a CFD study of their airway pressure and airflow velocity. CTL12 and CTL34 were defined as airflow velocity <3 and ≥3 m/s, respectively, and airway pressure <10 and ≥10 pa, respectively, in the narrowest upper airway.

**Results:**

Sixty-two patients (42 male and 20 female; mean age: 50.4 ± 14.6 years) were included; 12 and 50 patients had mild OSA and moderate to severe OSA, respectively. The minimum sPO2 in the supine position was 80.7 ± 9.1%. The total time of sPO2 <90% at overnight oximetry was 29.3 ± 51.1 min. Most (85.5%) neck CT examinations with CFD study presented CTL34. Patients with CTL34 had a lower minimum sPO2 in the supine position (78.4 vs. 88.1%, *P* = 0.004) and longer duration of sPO2 <90% at overnight oximetry (33.9 vs. 1.9 min, *P* = 0.001) than those with CTL12. The values of the area under the receiver operating characteristic curves of airway pressure and of airflow velocity at the narrowest upper airway were 0.788 and 0.733, respectively.

**Conclusion:**

Neck CT with CFD study of airway pressure and airflow velocity may provide a quick prediction of moderate to severe OSA.

## Introduction

Obstructive sleep apnea (OSA) is a so-called invisible killer disorder characterized by repetitive pauses of airflow through the upper airway with hypoxia during sleep (1). Intermittent hypoxemia may lead to systemic inflammation, which may play a vital role for risk of cardiovascular disease (CVD), diabetes, and cancer (1–4). A Swiss study observed that the prevalence of moderate to severe sleep apnea (apnea-hypopnea index [AHI] ≥ 15 episodes per hour) was 49.7% in men and 23.4% in women (5). Evidence has indicated that approximately 1 billion adults worldwide aged between 30 and 60 years may have OSA, which carries risks of major morbidity and mortality (2–4, 6). Patients with OSA may experience considerably worse health and quality of life (7).

The reference standard for diagnosis of OSA is a full-night polysomnography, a level 1 study, in a sleep center. However, patients with OSA experience substantial wait time (2–36 months) for sleep studies because OSA has become increasingly prevalent and diagnostic capacity remains limited (8–11). A full polysomnographic examination in a sleep center features data collection using electroencephalography, electrocardiography, electrooculography, chin and leg electromyography, thoracoabdominal bands, and snoring sensors and regarding body position, airflow, and saturation of pulse oxyhemoglobin (sPO2) (11). Therefore, many patients take sleeping pills to help overcome distraction and achieve sleep in their study. However, these medications may affect the sleep stages. Benzodiazepines may produce an increase and decrease in polysomnography readings at stage 2 and stage 3 sleep, respectively (12). Clinicians should be cognizant of a patient's medication before making a diagnosis in the sleep study.

The pathogenesis of OSA may result from pharyngeal collapse during sleep. Pharyngeal collapse can occur at the retropalatal level (uvula), the retroglossal level (tongue base), and at the oropharyngeal lateral walls and the hypopharynx (epiglottis) (13). Identification of the upper airway obstruction site is essential for an effective and nuanced treatment of OSA; current polysomnography for determining AHI cannot provide such necessary anatomic information. Although drug-induced sleep endoscopy (DISE) is an alternative modality for examining the obstruction site in patients with OSA, DISE requires sedation and may result in aspiration, laryngospasm, and deep desaturation (14, 15). Multidetector neck CT is a fast modality for scanning. Castro et al. (16) used computational fluid dynamics (CFD) simulations to construct the complex 3-dimensional airflow pattern in the human nasal passageways using the CT scan. Yu et al. (17) used neck CT with 3-dimensional models and reported substantial difference between the minimum cross-sectional area and the pressure gradient of the upper airway among patients with OSA and patients without OSA. Chousangsuntorn et al. reported that the presence of complete obstruction and complete concentric collapse in neck CT were associated with increased AHI for patients with OSA (18). Our study used a multidetector neck CT to reconstruct a 3-dimensional model of upper airway geometry as well as utilize CFD computations to evaluate airflow velocity and airway pressure of the upper airway. Relevant studies have indicated that having untreated moderate to severe OSA is correlated with increased cardiovascular morbidity and mortality (19–21). Therefore, we further used the results of the CFD study of airflow velocity and airway pressure to predict the moderate to severe OSA.

## Materials and Methods

### Study Participants

The patients in the study were diagnosed as having OSA (AHI ≥ 5 episodes per hour) with overnight oxygen desaturation (sPO2 <90%) in a full-night level-1 polysomnography session in a sleep center of the Taichung Hospital from February 1, 2020, to June 30, 2021. All patients received awake neck CT to evaluate the obstruction level of the upper airway from the skull base to the thoracic inlet by using a 160-slice Toshiba AQUILION PRIME scanner (Canon Medical Systems, Otawara-Shi, Tochigi-ken, Japan) with the following settings: 120 kVp, automatic mA, 1-mm slice thickness, 5-mm reconstruction interval, 1:1 helical pitch, and 30-cm display field of view with an approximately 5-s scan time (approximately 5 mGy of radiation exposure). We further constructed a 3-dimensional mesh model and geometry of upper airway from CT Digital Imaging and Communications in Medicine (DICOM) (Innolitics, Austin, TX, USA) images (image size 512 × 512). We excluded those patients who slept for <4 h. All personally identifiable information was digitally encrypted by a system used in the hospital. The study was approved by the Institutional Review Board of Tsaotun Psychiatric Center, Ministry of Health and Welfare (110008).

### Construction of Geometry and Mesh

**Figure 1:**
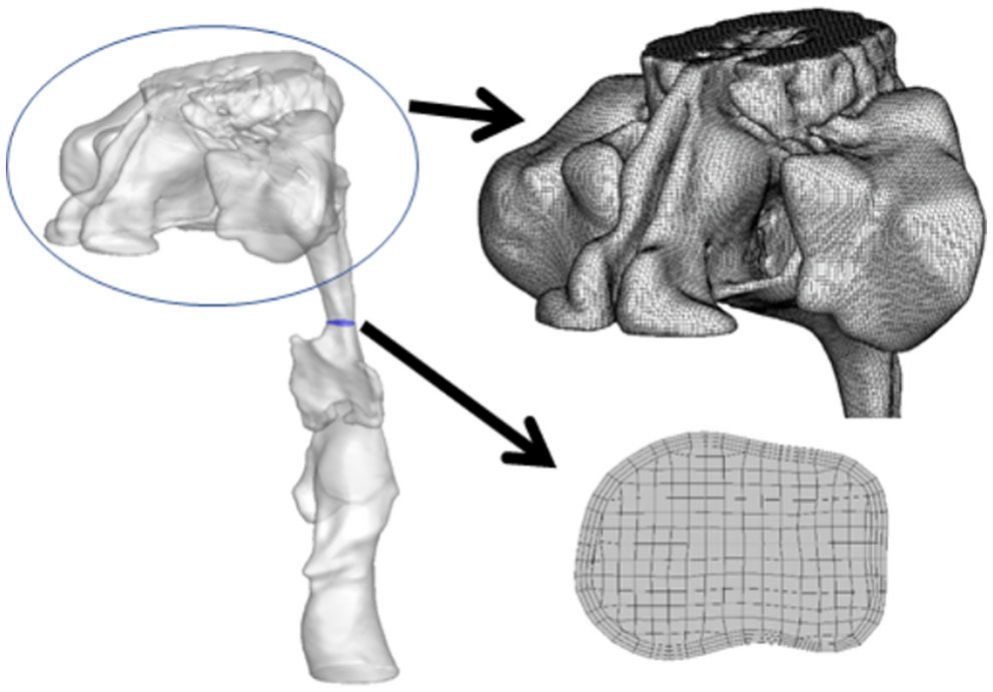
Geometry of upper airway and corresponding computational mesh arrangement.

The generation of an unstructured surface mesh, which was then saved in a stereolithography file, required the delineation of the upper airway and importation of the images into Soteria OPZ (Soteria Biotech, Taipei, Taiwan) and further construction of CFD airflow simulation (Figure 1). The unstructured tetrahedral meshes on the surface, structured hexahedral meshes in the core of the upper airway, and pentahedral meshes inside the transition region were generated by snappyHexMesh software (OpenFOAM, London, UK).

### Numerical Model and Method

The 3-dimensional incompressible flows in turbulence were determined by the Reynolds-averaged Navier–Stokes equations (RANS) inside the upper airway. The gravitational effect, heat source, heat transfer, phase change, and chemical reactions were disregarded. Turbulence closure was obtained using the standard shear stress transport k–ω model (22). The velocity profile as a function of time (*t*) was imposed at the inlet of the airway as


(1) $$\begin{array}{l} U=U_{i}\sin\left(2\pi ft\right) \end{array}$$

where Ui and f are the amplitude and frequency, respectively, of the variation in velocity (17). The transient calculation of the upper airway included four cycles of respiration.

The density and dynamic viscosity of inhaled fluid were set at 1.1614 kg/m3 and 1.846e−5 kg/ms, respectively, because we expected normal air inhalation from the nose. The inlet boundary was located at the nasopharynx and the velocity of the flow was variably set to represent normal breathing; the outlet boundary was positioned at the hypopharynx where the pressure was set at 0 Pa with the reference pressure of 1 atm. A no-slip condition was imposed on the surface of the trachea. Null initial velocity and pressure were set in the trachea. The simulation condition involved a breathing period (T = 1/f) of 3.75 s and a tidal volume of 500 ml. The RANS was solved using the finite volume method in Soteria OPZ software.

### Working Definition of CFD Study

Airflow velocity was used to indicate a normal (<1 m/s), mild severity (1–3 m/s), moderate severity (3–5 m/s), or severe severity (>5 m/s) condition. Airway pressure was used to indicate a normal (<3 pa), mild severity (3–10 pa), moderate severity (10–20 pa), or severe severity (>20 pa) condition. We defined CTL12 and CTL 34 as airflow velocity <3 and ≥3 m/s, respectively, and airway pressure <10 and ≥10 pa, respectively, in the narrowest upper airway. We evaluated airway pressure and airflow velocity by using area under the receiving operating characteristic curve (AUROC) to predict moderate to severe OSA.

### Statistical Analyses

We conducted statistical analyses by using SPSS Version 22.0 (IBM, Armonk, NY, USA). The Chi-square test was used to compare and test the differences in categorical variables between both groups. The Mann–Whitney *U* test was used to compare the continuous variables of both groups. The *P* < 0.05 was represented as a statistical significance level for all tests. We also computed the AUROC for a determination of prediction discrimination of airway pressure and airflow velocity for diagnosing moderate to severe OSA.

## Result

The participants were 70 patients with OSA and overnight oxygen desaturation who received awake neck CT with a CFD study of airflow velocity and airway pressure. We excluded 8 patients who slept for <4 h. No substantial differences between demographics and OSA severity were present between the individuals included and excluded. The remaining 62 patients (42 male and 20 female) were included in the analyses. Most of the study patients (51, 82.3%) were never smokers. Seven patients were current smokers and four patients were ex-smokers. Their mean age was 50.4 ± 14.6 years. Their body mass index (BMI), neck circumference, and waist circumference were 27.6 ± 4.0 kg/m^2^, 38.5 ± 3.5 cm, and 96.9 ± 8.7 cm, respectively. The average AHI was 34.7 ± 24.1 episodes per hour. In total, 12 patients with mild OSA and 50 patients with moderate to severe OSA participated. The minimum sPO2 of overnight oximetry was 80.3 ± 8.9%. The minimum sPO2 in the supine position was 80.7 ± 9.1%. The total duration of sPO2 <90% at overnight oximetry for each participant was 29.3 ± 51.1 min. The mean airway pressure was 40.3 ± 55.9 Pa and the mean airflow velocity was 9.7 ± 7.3 m/s. Most (85.5%) neck CT examinations with CFD study were of CTL34 (Table 1).

**Table 1:** Demographic characteristics of study participants.

**Variables**	***N* = 62**	
	***N* (mean)**	**% (SD)**
**Sex**		
Male	42	67.7
Female	20	32.3
Age	(54.0)	(14.6)
Epworth sleepiness scale	(8.1)	(4.3)
Berlin questionnaire high risk	17	29.8
Body mass index (kg/m^2^)	(27.6)	(4.0)
Neck circumferences (cm)	(38.5)	(3.5)
Waist circumferences (cm)	(96.9)	(8.7)
AHI (/h)	(34.7)	(24.1)
Mild severity of OSA	12	19.4
Moderate to severe severity of OSA	50	80.6
Minimum sPO2 of overnight oximetry (%)	(80.3)	(8.9)
Minimum sPO2 in supine position (%)	(80.7)	(9.1)
sPO2 <90% (min) at overnight oximetry	(29.3)	(51.1)
N1 + N2 phase of TST (%)	(70.6)	(13.0)
N3 phase of TST (%)	(9.7)	(8.4)
Airway pressure (pa)	(40.3)	(55.9)
Airflow velocity at the narrowest upper airway (m/s)	(9.7)	(7.3)
CTL12	9	14.5
CTL34	53	85.5

*SD, standard deviation; AHI, apnea-hypopnea index; REM, rapid eye movement; RDI, respiratory disturbance index; sPO2, pulse oxygen saturation; PSG, polysomnography; TST, total sleep time; CTL12, airflow velocity <3 m/s and airway pressure <10 Pa in the narrowest upper airway by neck CT; CTL34, Airflow velocity ≥ 3 m/s or airway pressure ≥10 Pa in the narrowest upper airway by neck CT*.

Table 2 presents the results of patients with mild OSA and those with moderate to severe OSA for comparison. Patients with mild OSA and those with moderate to severe OSA did not substantially differ with respect to BMI, neck circumference, or waist circumference. The patients with moderate to severe OSA exhibited considerably less deep sleep (8.2 vs. 16.2%, *P* = 0.004) and more shallow sleep (72.2 vs. 63.8%, *P* = 0.023) than did the patients with mild OSA. Furthermore, patients with moderate to severe OSA had substantially lower sPO2 (78.8 vs. 88.7%, *P* < 0.001) and a longer duration of sPO2 <90% at overnight oximetry (36.2 vs. 0.4 min, *P* < 0.001) than did the patients with mild OSA. The patients with moderate to severe OSA carried substantially higher airway pressure (47.0 vs. 13.0 pa, *P* = 0.005) and airflow velocity (10.7 vs. 5.9 m/s, *P* = 0.023) in the narrowest upper airway than did the patients with mild OSA.

**Table 2:** Results for patients with mild OSA and patients with moderate to severe OSA.

**Variables**	**Mild OSA** **(*N* = 12)** **M ±SD (*n*, %)**	**Moderate to severe** **OSA (*N* = 50)** **M ±SD (*n*, %)**	***P*-value**
Male	(6, 14.3%)	(36, 85.7%)	0.177
Age (y)	46.2 ± 18.3	55.9 ± 13.1	0.066
ESS	8.5 ± 5.1	8.0 ± 4.1	0.95
Berlin questionnaire high risk	(2, 18.2%)	(15, 32.6%)	0.476
BMI (kg/m^2^)	27.1 ± 4.2	27.8 ± 3.9	0.575
Neck circumference (cm)	38.0 ± 4.2	38.6 ± 3.4	0.430
Waist circumference (cm)	94.4 ± 9.2	97.5 ± 8.6	0.378
RDI in REM phase (/h)	18.0 ± 11.8	50.8 ± 21.1	<0.001
RDI in NREM phase (/h)	6.4 ± 3.0	38.3 ± 24.5	<0.001
Minimum sPO2 at overnight oximetry (%)	88.1 ± 2.0	78.4 ± 8.9	<0.001
Minimum sPO2 in supine position (%)	88.7 ± 1.9	78.8 ± 9.1	<0.001
Total time of sPO2 <90% (min) at overnight oximetry	0.4 ± 0.5	36.2 ± 54.8	<0.001
N1+N2 phase of TST (%)	63.8 ± 11.6	72.2 ± 12.9	0.023
N3 phase of TST (%)	16.2 ± 9.9	8.2 ± 7.3	0.004
Airway pressure (pa)	13.0 ± 8.3	47.0 ± 60.5	0.005
Airflow velocity at the narrowest upper airway (m/s)	5.9 ± 3.9	10.7 ± 7.6	0.023

*OSA, obstructive sleep apnea; Mann–Whitney U test.*

*M, mean; SD, standard deviation; ESS, Epworth sleepiness scale; BMI, body mass index; REM, rapid eye movement; RDI, respiratory disturbance index; sPO2, pulse oxygen saturation; PSG, polysomnography; TST, total sleep time*.

**Figure 2:**
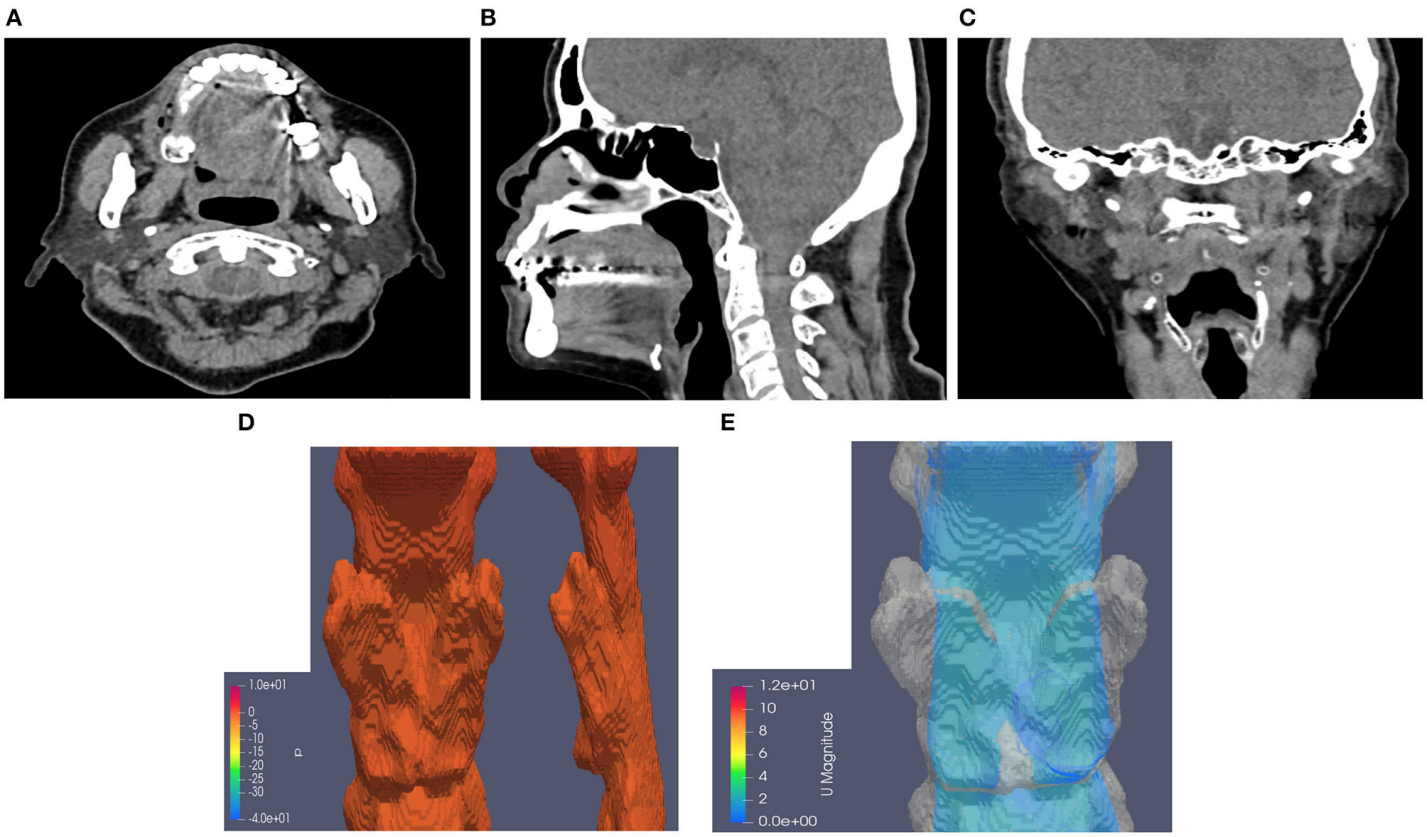
Neck CT with computational fluid dynamics study of the 82-year-old female showed CTL12. **(A)** Axial view. **(B)** Sagittal view. **(C)** Coronal view. **(D)** Airway pressure of 1.28 Pa. **(E)** Airflow velocity of 0.8 m/s.

**Figure 3:**
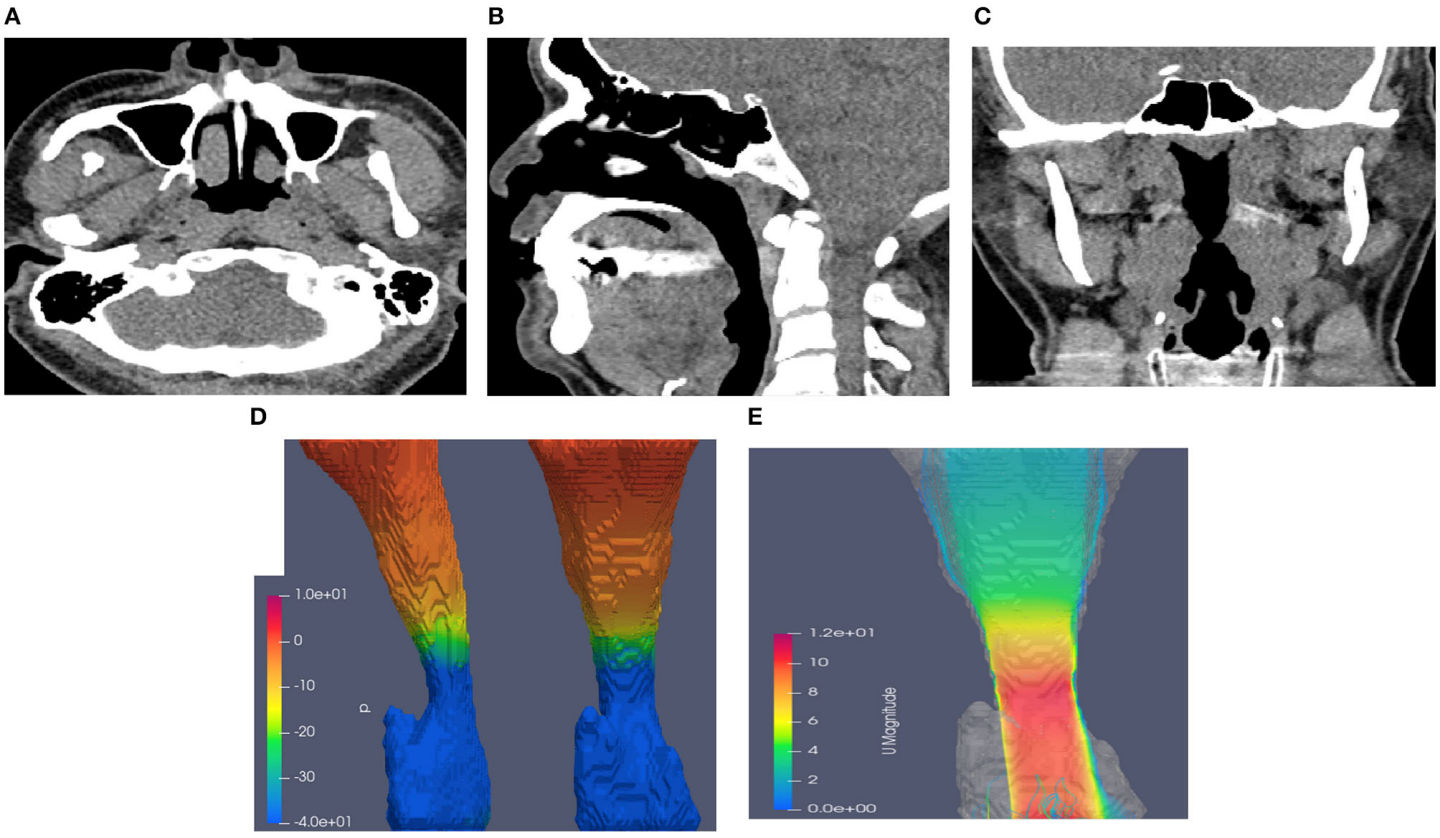
Neck CT with computational fluid dynamics study of the 44-year-old male showed CTL34. **(A)** Axial view. **(B)** Sagittal view. **(C)** Coronal view. **(D)** Airway pressure of 51.88 Pa. **(E)** Airflow velocity of 10.7 m/s.

Table 3 presents the results of CTL12 and CTL34 for comparison. The patients with CTL34 exhibited a larger waist circumference (97.9 vs. 90.8 cm, *P* = 0.037) and higher AHI (38.0 vs. 16.1 per hour, *P* = 0.003) than did the patients with CTL12. The patients with CT34 had lower minimum sPO2 in the supine position (78.4 vs. 88.1%, *P* = 0.004) and longer duration of sPO2 <90% at overnight oximetry (33.9 vs. 1.9 min, *P* = 0.001) than did the patients with CTL12. We illustrated neck CT with CFD study for CTL12 in the (Figure 2) and for CTL34 in the (Figure 3).

**Table 3:** Results for patients with CTL12 or CTL34 as determined using awake neck CT with CFD study.

**Variables**	**CTL12** **(*N* = 9)** **M ±SD (*n*, %)**	**CTL34 (*N* = 53)** **M±SD (*n*, %)**	***P*-value**
Male	(6, 14.3%)	(36, 85.7%)	0.941
Age (y)	59.3 ± 15.5	53.1 ± 14.4	0.142
BMI (kg/m^2^)	25.2 ± 3.6	28.0 ± 3.9	0.060
Neck circumference (cm)	38.1 ± 3.9	38.6 ± 3.5	0.430
Waist circumference (cm)	90.8 ± 7.9	97.9 ± 8.5	0.037
AHI (/h)	16.1 ± 12.6	38.0 ± 23.9	0.003
RDI in REM phase (/h)	25.0 ± 15.7	47.8 ± 23.1	0.007
RDI in NREM phase (/h)	13.1 ± 13.7	35.4 ± 25.6	0.003
Minimum sPO2 at overnight oximetry (%)	88.1 ± 2.0	78.4 ± 8.9	0.004
Minimum sPO2 in supine position (%)	88.7 ± 1.9	78.8 ± 9.1	0.012
Total time of sPO2 <90% (min) at overnight oximetry	1.9 ± 3.2	33.9 ± 54.0	0.001
N1+N2 phase of TST (%)	71.5 ± 14.6	70.4 ± 12.9	0.772
N3 phase of TST (%)	10.1 ± 8.4	9.7 ± 8.5	0.803

*CT, computed tomography; CFD, computational fluid dynamics; Mann–Whitney U test; CTL12, airflow velocity <3 m/s and airway pressure <10 Pa in the narrowest upper airway; CTL34, Airflow velocity ≥3 m/s or airway pressure ≥10 Pa in the narrowest upper airway; M, mean; SD, standard deviation; BMI, body mass index; AHI, apnea-hypopnea index; REM, rapid eye movement; RDI, respiratory disturbance index; sPO2, pulse oxygen saturation; PSG, polysomnography; TST, total sleep time*.

**Figure 4:**
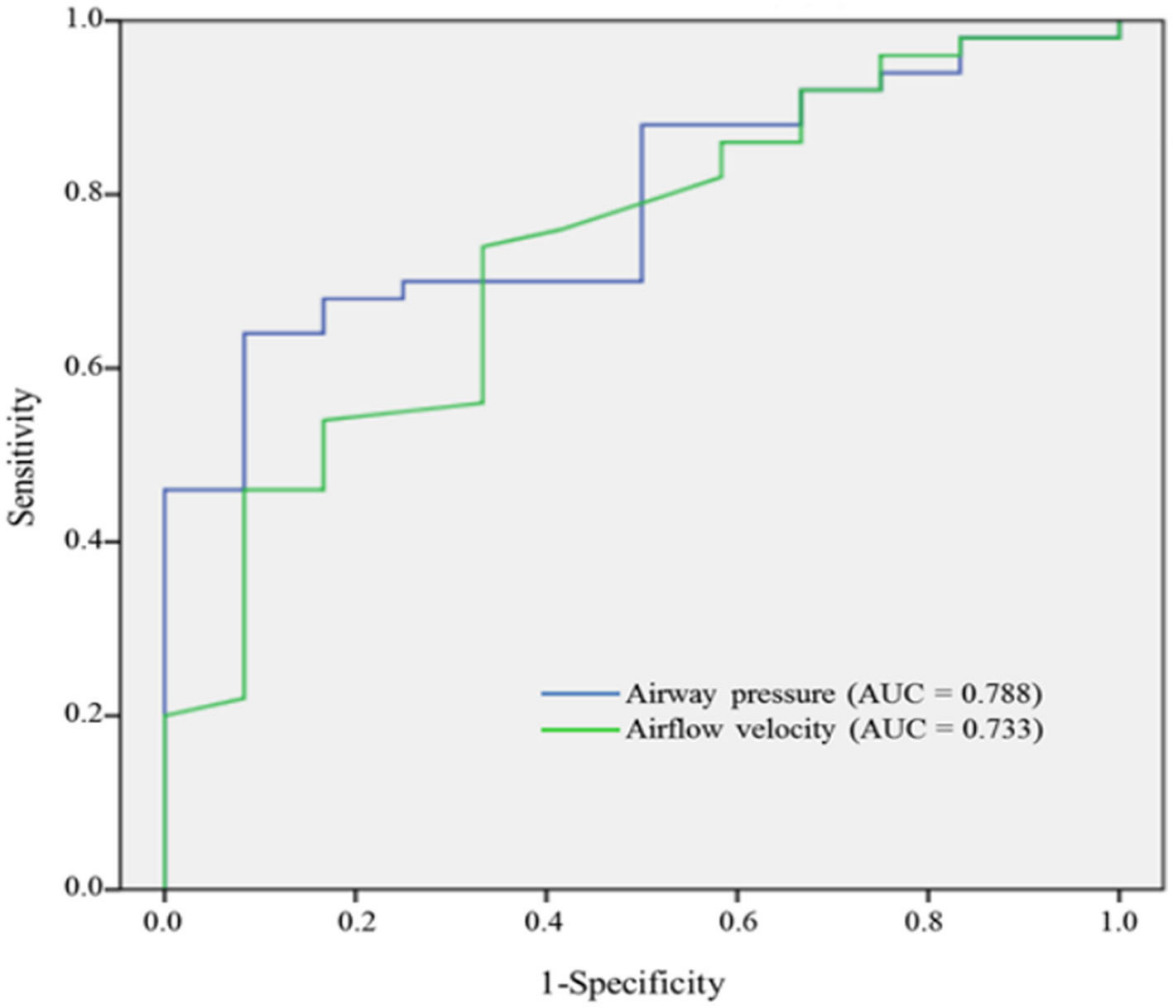
AUROC curves of airway pressure and airflow velocity for predicting moderate to severe OSA.

As depicted in (Figure 4), the values of the AUROC for airway pressure and airflow velocity at the narrowest upper airway for predicting moderate to severe OSA were 0.788 and 0.733, respectively. Our definition of CTL34 for the prediction discrimination of moderate to severe OSA yielded a sensitivity of 92%, specificity of 42%, positive predictive value of 87%, and negative predictive value of 56%.

## Discussion

To the best of our knowledge, this is the first study to investigate the effectiveness of using awake neck CT with a CFD study of both airway pressure and airflow velocity to predict moderate to severe OSA. Airway pressure and airflow velocity through a CFD study of neck CT at the narrowest upper airway provided acceptable discrimination for moderate to severe OSA (AUROC = 0.788 for airway pressure and AUROC = 0.733 for airflow velocity, respectively). Concomitant airway pressure and airflow velocity provided a sensitivity of 92% and a specificity of 42%. Awake neck CT provides a quick screening modality not only for moderate to severe OSA but also for the anatomic obstruction site of the upper airway. However, awake CT may not completely reveal upper airway collapse caused by decreased muscle tone during sleep, and this limitation may result in relatively low specificity. Albeit previous studies indicated that Berlin questionnaire and Epworth sleepiness scale may be helpful to screen OSA (23, 24). However, neither Berlin questionnaire nor Epworth sleepiness scale can predict moderate to severe OSA in the current study.

Barkdull et al. reported that patients with OSA exhibited smaller cross-sectional areas in the retroglossal airway and a larger distance between the mandible and hyoid (25). Although AHI is correlated with the retroglossal airway and distance between the mandible and hyoid, a clear threshold is not available for demarcating moderate to severe OSA. Chousangsuntorn et al. suggested that patients with severe OSA were likely to have complete concentric collapse of the upper airway in neck CT examination after they took 10 mg of zolpidem hemitartrate (18). Our study participants received awake neck CT examinations without the use of sedatives because the overall examination periods lasted only approximately 5 min. Furthermore, we utilized CFD computations to calculate concomitant airflow velocity and airway pressure at the narrowest upper airway, which indicated acceptable predictors of moderate to severe OSA.

Although overnight polysomnography in a sleep center is considered a diagnostic standard for OSA (26), the method is time consuming and cannot determine the anatomic obstruction site of the upper airway. BMI, neck circumference, and waist circumference were not found to be factors contributing to mild OSA and moderate to severe OSA in our study. Table 2 demonstrates that the patients with moderate to severe OSA exhibited substantially lower overnight minimum sPO2 and longer overnight hypoxemia than did the patients with mild OSA. Overnight hypoxemia may result in oxidative stress, systemic and vascular inflammation, vasoconstriction, and endothelial dysfunction, which become pivotal risk factors that contribute to the development of OSA-related comorbidities (27). Patients with OSA with hypoxemia carry risk of developing not only comorbid CVD but also worsened CVD-related outcomes (28). Thus, we selected patients diagnosed with OSA with overnight oxygen desaturation and searched for a potential quick-examination modality.

We further evaluated patients by using neck CT with a CFD study of airway pressure and airflow velocity and categorized the readings as indicating CTL12 or CTL34. The patients with CTL34 exhibited a larger waist circumference than did the patients with CTL12. Waist circumference was strongly correlated with total amount of visceral fat, which may result in increased adipose tissue within the airway and a reduced upper airway size (29, 30). Table 3 demonstrates that the patients with CTL34 had substantially decreased minimum sPO2 in the supine position, increased AHI, and prolonged overnight hypoxemia compared with the patients with CTL12. The patients in the awake neck CT with CFD study that exhibited airflow velocity ≥ 3 m/s or airway pressure ≥ 10 Pa are highly suggestive of moderate to severe OSA and require prompt treatment. Untreated OSA with overnight hypoxemia increases risks of CVD and mortality (28).

A primary strength of this study is that it is the first to conduct an awake neck CT with CFD study of airway pressure and airflow velocity that provides acceptable diagnosis of moderate to severe OSA. This study's findings suggest that awake neck CT shortens examination wait time (~5-s scan time) and reduces the need for sedative medications. Relevant studies have used DISE to evaluate the upper airway condition of patients with OSA (31, 32). Both sedative medication and the depth of sedation simulating natural sleep should be considered. However, several limitations must be considered when interpreting these findings. First, repetitive episodes of upper airway collapse during sleep may be masked by awake neck CT. This can explain why a CFD study of airway pressure and airflow velocity by awake neck CT provides relatively low specificity. Additionally, the patients with overnight oxygen desaturation must receive a comprehensive sleep study even if awake neck CT with CFD study of airway pressure and airflow velocity did not meet the criteria of CTL34. Second, this retrospective study might have produced biased results (e.g., OSA prevalence) because the data were collected from a single sleep center.

## Conclusion

This study reports that neck CT with a CFD study of airway pressure and airflow velocity may provide an acceptable prediction of moderate to severe OSA. Moreover, neck CT can shorten diagnostic examination wait time and provide good measurements of upper airway anatomy.

## Data Availability Statement

The original contributions presented in the study are included in the article/supplementary material, further inquiries can be directed to the corresponding author.

## Ethics Statement

The studies involving human participants were reviewed and approved by the Institutional Review Board of Tsaotun Psychiatric Center, Ministry of Health and Welfare (110008), Taiwan. Written informed consent for participation was not required for this study in accordance with the national legislation and the institutional requirements.

## Author Contributions

W-SC: conception and design and administrative support. All authors collected, assembly of data, data analysis, interpretation, manuscript writing and final approval of manuscript.

## Funding

This study was supported by Taichung Hospital, Ministry of Health and Welfare (TAIC110-01). The funders had no role in study design, data collection and analysis, decision to publish, or preparation of the manuscript.

## Conflict of Interest

The authors declare that the research was conducted in the absence of any commercial or financial relationships that could be construed as a potential conflict of interest.

## Publisher's Note

All claims expressed in this article are solely those of the authors and do not necessarily represent those of their affiliated organizations, or those of the publisher, the editors and the reviewers. Any product that may be evaluated in this article, or claim that may be made by its manufacturer, is not guaranteed or endorsed by the publisher.
